# Understanding cholera dynamics in African countries with persistent outbreaks: a mathematical modeling approach

**DOI:** 10.1186/s12889-025-24507-0

**Published:** 2025-10-08

**Authors:** Adeniyi Ebenezer, Sarafa Adewale Iyaniwura, Andrew Omame, Qing Han, Xiaoying Wang, Nicola L. Bragazzi, Woldegebriel Assefa Woldegerima, Jude D. Kong

**Affiliations:** 1 Department of Mathematics and Statistics, York University, Toronto, Ontario Canada; 2 Biostatistics, Bioinformatics, and Epidemiology Program, Fred Hutchinson Cancer Center, Seattle, WA 98109 USA; 3 Africa-Canada Artificial Intelligence and Data Innovation Consortium (ACADIC), Toronto, Ontario Canada; 4 Department of Mathematics & Statistics, Trent University, Peterborough, Ontario Canada; 5 Department of Clinical Pharmacy, Saarland University, 66123 Saarbrücken, Germany; 6 Department of Food and Drugs, University of Parma, 43125 Parma, Italy; 7 United Nations Educational, Scientific and Cultural Organization (UNESCO), Health Anthropology Biosphere and Healing Systems, University of Genoa, 16126 Genoa, Italy; 8 Artificial Intelligence & Mathematical Modeling Lab (AIMM Lab), Dalla Lana School of Public Health, University of Toronto, 155 College St Room 500, Toronto, Ontario M5T 3M7 Canada; 9 Department of Mathematics, University of Toronto, Toronto, Ontario Canada; 10 Global South Artificial Intelligence for Pandemic and Epidemic Preparedness and Response Network (AI4PEP), Toronto, Ontario Canada; 11 Department of Mathematics, Federal University of Technology, Owerri, Nigeria; 12 Department of Mathematics, CNCS, Mekelle, Tigray Ethiopia

**Keywords:** Cholera transmission, Modified iSIRB models, Infectious disease modeling, Basic reproduction number, Bayesian parameter estimation, Sensitivity analysis, Hierarchical clustering, Public health surveillance

## Abstract

**Background:**

Cholera, caused by *Vibrio cholerae*, is a global health challenge, spreading through water in areas lacking clean water and sanitation. Since 2021, the reemergence of cholera cases has increased significantly in endemic regions in Africa. In particular, the continent experienced severe outbreaks between 2022 and 2024 due to droughts and cyclones, which have placed additional strain on healthcare systems.

**Objective:**

This study aims to investigate the dynamics of cholera outbreaks in eight African countries using mathematical modeling and machine learning and to provide information for public health decision making. By estimating key model parameters and epidemiological indicators, such as the basic reproduction number, we aim to identify and quantify the impacts of key transmission drivers. Using this together to socioeconomical factors, we will be classifying cholera persistent countries with similar dynamics using unsupervised learning. In addition, the study seeks to provide information on cholera outbreaks and management across the selected countries, identify key drivers of outbreak intensity, and propose targeted intervention strategies.

**Methods:**

A compartmentalized epidemiological model with indirect transmission routes is analyzed for cholera dynamics in eight African countries with persistent outbreaks. The key parameters and initial values of the model’s variables were estimated using a Bayesian framework. We assessed some outcomes such as the reproduction number, “$\mathcal {R}_0$," outbreak peak duration and size. Moreover, environmental and socioeconomic data were used in hierarchical clustering to group countries by outbreak characteristics.

**Results:**

The study uncovered variation in cholera outbreak dynamics across the considered countries. Based on our model results, the median basic reproduction number ($\mathcal {R}_0$) across the endemic countries was 2.0 (*SD* : 0.454), which ranges from 1.41 in Zimbabwe to 2.80 in Mozambique. Furthermore, the results of the sensitivity analysis emphasized the significance of the maximum infection rate and the bacteria shedding rate in driving cholera outbreaks across the endemic regions in Africa. Hierarchical clustering revealed three distinct groups of countries based on outbreak dynamics and socioeconomic indicators: the chronic sanitation issues cluster (Somalia, Cameroon, and Comoros); the economic and infrastructure challenges cluster (Sudan, Zimbabwe, and Zambia); and the natural disaster cluster (Malawi and Mozambique).

**Conclusion:**

This study highlights the drivers of cholera outbreaks across African countries, emphasizing the need for tailored interventions that consider underlying socio-demographic and environmental vulnerabilities. The findings underscore the importance of integrating data-driven approaches into cholera preparedness and response efforts to mitigate its impact.

**Supplementary Information:**

The online version contains supplementary material available at 10.1186/s12889-025-24507-0.

## Introduction

Cholera remains a significant global health threat, particularly in regions with inadequate access to potable water, hygiene, and sanitation infrastructure. The etiological agent of cholera, *Vibrio cholerae*, is a facultative anaerobe found in contaminated water sources, typically propagated by the fecal-oral route [49]. Upon ingestion, *V. cholerae * colonizes the small intestine and secretes cholera toxin (CT), a potent enterotoxin that induces profuse, watery diarrhea, the hallmark symptom of the disease [9]. If left untreated, the resultant dehydration and electrolyte imbalance can rapidly lead to hypovolemic shock and death [54]. The incubation period for cholera varies from 12 hours to five days post-exposure [21]. In severe cases, metabolic acidosis ensues, contributing to high mortality rates, often within 24 hours of symptom onset ([12, 30]). Notably, in endemic areas, characterized by reinfections and multiple outbreaks, individuals who recover from infection develop a degree of acquired immunity, although the duration and efficacy of this immune protection remain subject to host and environmental factors [18].

The historical significance of cholera as a global pathogen is underscored by its Pandemic occurrences, beginning with the first Pandemic in 1817. The seminal epidemiological work of Dr. John Snow during the London outbreak of 1854 identified contaminated drinking water as the primary vector of transmission, pioneering modern public health interventions [1]. To date, seven cholera pandemics have been recorded, each contributing to substantial morbidity and mortality worldwide [10]. The transmission pathways of cholera are primarily mediated through aquatic reservoirs, exacerbated by human activities and climatic fluctuations [40, 41]. Additionally, person-to-person transmission has been documented, highlighting the necessity for stringent public health measures to curb outbreaks [16]. In endemic areas, where re-infection occurs, the persistence of *V. cholerae* in the environment is facilitated by asymptomatic carriers who shed bacteria, further propagating its endemicity in vulnerable regions. However, in this study, we focus on the recent epidemic outbreaks in Africa [37].

Since the onset of the seventh cholera Pandemic in 1961, an estimated 2.9 million cases and 95,000 deaths occur annually [46]. The burden of disease is disproportionately concentrated in Africa and Asia, with Africa alone accounting for over 80% of global cholera cases due to compounded factors such as conflict-induced displacement, natural disasters, and infrastructural deficiencies [29]. As of February 2023, cholera outbreaks had been reported in 25 countries, with the highest incidence in South Asia and sub-Saharan Africa. Between January and August 2023, 14 African nations, including Nigeria, Cameroon, the Democratic Republic of the Congo, South Sudan, and Ethiopia, reported significant case surges, contributing to a cumulative total of 547,626 laboratory-confirmed cases and 4,927 fatalities [13]. The intersection of environmental catastrophes and cholera epidemiology is well-documented, with major outbreaks following extreme weather events such as the Mozambique floods of 2000 and the volcanic eruption in the Democratic Republic of the Congo in 2002 [36]. Moreover, climate variability, particularly El Niño events, has been linked to exacerbated cholera transmission, with an estimated excess burden of 50,000 cases in East Africa during El Niño-associated anomalies [31].

To mitigate the cholera burden, the World Health Organization (WHO) advocates a multifaceted intervention strategy encompassing water quality improvement, sanitation infrastructure, hygiene promotion, medical management, and oral cholera vaccination [39]. This integrated approach has been widely adopted by public health agencies and non-governmental organizations. In Malawi, for instance, the confluence of cholera and other public health crises, such as polio outbreaks and tropical storms, has necessitated an aggressive WASH (Water, Sanitation, and Hygiene) intervention framework. Between 2022 and 2023, Malawi reported over 59,000 cholera cases and 1,770 deaths. However, sustained WASH initiatives and enhanced surveillance systems facilitated a decline in cases in 2023 [28]. Despite these efforts, the persistence of cholera in Africa underscores the critical need for sustained investment in infrastructure, vaccine coverage, and rapid response mechanisms.

Mathematical modeling can serve as a crucial tool in understanding and predicting cholera transmission dynamics, thereby informing public health policies and strategic interventions [4]. The choice of modeling framework depends on the nature of the pathogen’s transmission mechanisms. The classical Susceptible-Infected-Recovered (SIR) model, first introduced by Kermack and McKendrick in 1927 [22], provides a fundamental structure for epidemiological modeling. However, cholera’s indirect transmission dynamics necessitate an extended framework incorporating environmental reservoirs. The seminal work of Capasso and Fontana (1979) introduced a nonlinear incidence function to better represent *V. cholerae* transmission [6], laying the foundation for contemporary cholera modeling approaches.

Recent advances in understanding the complex dynamics of cholera transmission have increasingly emphasized the importance of integrating environmental data, vaccination strategies, and various control measures into infectious disease modeling frameworks. In a model proposed by Brhane et al. (2024) [5], key parameters such as the intrinsic growth rate of the pathogen, vaccination coverage, water sanitation, and therapeutic water treatment rates were found to significantly influence the basic reproduction number. The study demonstrated that increasing the rate of intervention exerts a suppressive effect on the basic reproduction number, and that sustained vaccination, in combination with improved treatment and sanitation measures, can effectively reduce the spread of infection. Complementing this perspective, Mushanyu et al. (2024) [34] developed a cholera transmission model that incorporates antibiotic resistance and stage-switching treatment dynamics for patients transitioning to second-line therapies following treatment failure. Their findings highlight antibiotic-resistant cholera as a major driver of overall transmission. Sensitivity analyses revealed that parameters associated with the resistant strain exert the strongest influence on transmission dynamics. These insights underscore the critical need for targeted interventions aimed specifically at mitigating resistance-related transmission pathways.

In recent years, the use of machine learning (ML) techniques to complement traditional compartmental models has been on the rise for exploratory analysis, pattern recognition, and predictive tasks. Hierarchical clustering, a form of unsupervised ML, can provide a means of identifying spatial and temporal clusters of disease cases, discovering transmission hotspots, and stratifying regions based on risk profiles [26]. Integrating ML techniques with mechanistic models provides a more comprehensive and data-driven understanding of disease dynamics.

Current modeling approaches rely on classical compartmental models that focus only on human-to-human transmission. In this study, to understand the complex interplay of cholera dynamics not captured by classical epidemiological models, an indirectly transmitted SIRB (iSIRB) model (indirect transmission-susceptible-infected-recovered-bacteria) compartmental model was used to analyze cholera outbreaks in eight African nations between 2022 and 2024. This model explicitly accounts for indirect transmission via environmental contamination, incorporating epidemiological parameters such as bacterial shedding rates, minimum infectious dose, and pathogen decay rates. Parameter estimation was conducted using a Monte Carlo Markov Chain (MCMC) methodology to refine model accuracy. Hierarchical clustering techniques were applied to categorize countries based on cholera transmission profiles, potentially allowing for the implementation of targeted intervention strategies. Sensitivity analyses further elucidated the influence of critical parameters on outbreak dynamics, enhancing the robustness of predictive assessments. This modeling framework provides a comprehensive understanding of cholera epidemiology in African countries highly impacted by cholera and can inform strategic public health responses to mitigate future outbreaks.

## Methods

### Mathematical model

**Fig. 1:**
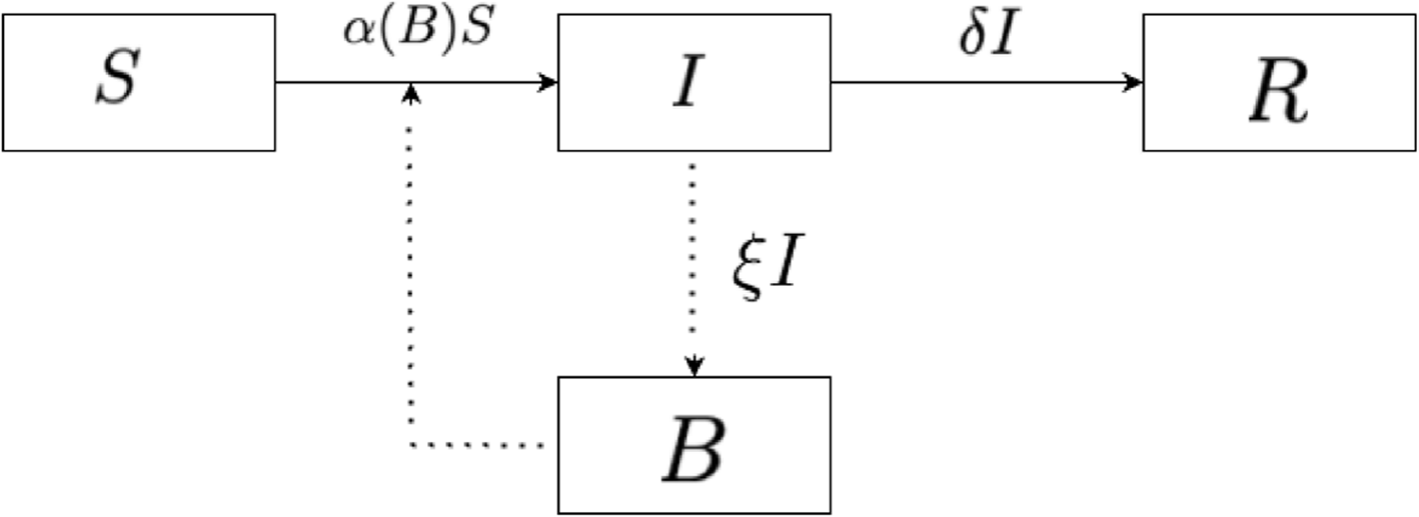
Schematic representation of the iSIRB model. Solid arrows indicate transitions between population compartments, while dashed arrows represent bacterial shedding into the environment and transmission through contaminated water

We adopt an iSIRB compartmental model [20, 32] to analyze the dynamics of cholera in eight cholera epidemic African countries. Although direct person-to-person transmission of cholera is possible, it remains relatively rare [16]. Instead, the primary transmission route in Africa is through the consumption of contaminated water [14]. Therefore, our model emphasizes indirect transmission via waterborne pathways. The iSIRB model consists of four compartments: three representing the dynamics of the human population and one tracking the bacterial concentration in water (see Fig. 1 and Table 1). The human compartments are: Susceptible (*S*): Individuals at risk of exposure to *V. cholerae* through contaminated water sources. Infected (*I*): individuals currently infected, with bloodstream bacterial concentrations exceeding the minimum infectious dose necessary to cause illness. Recovered (*R*): Individuals who have recovered from infection and are no longer actively shedding bacteria. For the considered period of the outbreak, these individuals acquired temporary immunity, which wanes only after the epidemic, which makes them not susceptible for the time period. The final compartment, *B*, represents the concentration of *V. cholerae* in the water reservoir, which serves as the primary vehicle for transmission. We assume the population of each region under consideration is constant, i.e. $N = S+I+R$ is constant.

**Table 1:** Model parameters and their descriptions

Parameter	Description	Dimension
*S*	Susceptible population	Persons
*I*	Infected population	Persons
*R*	Recovered population	Persons
*B*	Concentration of *Vibrio cholerae* in the reservoir (value = $10^6$)	$\text {Cell liter}^{-1}$
*N*	Total population	Persons

Since our model describes cholera transmission at a national scale, not every individual is equally exposed to contaminated water sources. We therefore assume that only a fraction of the total population is susceptible during an outbreak. This fraction is estimated using Bayesian inference.

Furthermore, the model assumes that the concentration of *V. cholerae* in a water reservoir is directly proportional to the bacterial load ingested through water consumption. Infection occurs only when the bacterial concentration exceeds a critical threshold—the minimum infectious dose. Infected individuals contribute to bacterial shedding through fecal contamination, which increases environmental *V. cholerae* levels [42]. This process sustains transmission by reintroducing bacteria into water sources, thereby perpetuating outbreaks.

The model differential equations are given by

1 $$\begin{aligned} \begin{array}{l} \frac{dS}{dt} = -\alpha (B)S, \\ \frac{dI}{dt} = \alpha (B)S - \mu I - \gamma I, \\ \frac{dR}{dt} = \gamma I, \\ \frac{dB}{dt} = rB \left( 1 - \frac{B}{K}\right) + \xi I, \\ \end{array} \end{aligned}$$

where $\alpha (B)$ is the cholera transmission incidence rate, given by

2 $$\begin{aligned} \alpha (B)=\left\{ \begin{array}{lr} 0 & B<c, \\ \dfrac{\eta (B-c)}{(B-c)+H} & B \ge c. \end{array} \right. \end{aligned}$$

Here, *c* is the minimum infectious dose of *V. cholerae* bacteria required to trigger an infection [32]. We observed from Eq. (2) that there is no cholera infection in the population when $B <c$, and the spread of the disease only occurs when the reservoir bacteria concentration is equal to or greater than the threshold (i.e., $B \ge c$), a condition that must be satisfied in the model.

In outbreak-prone regions, the *V. cholerae* is cultivable during the outbreak but disappears afterward. However, it has been shown that the bacteria enter a viable but non-culturable state after the outbreak subsides as its survival mechanism [15]. In this model, bacteria grow and multiply using logistic growth with a maximum carrying capacity (*K*), which is insufficient to cause an epidemic. External factors such as seasonal changes, climate variation, and conflicts can elevate the *V. cholerae* level beyond the maximum capacity, reaching the threshold required for an outbreak. At the start of an epidemic, it is necessary for the initial concentration of the bacteria, $B_0$, to be greater than or equal to its maximum capacity and more importantly greater than the minimum infection dose (*c*) for an outbreak to occur.

**Table 2:** Model parameters used in the iSIRB framework, including descriptions, units, and value types. Fixed parameter values were based on prior studies such as [23] and [35]

Parameter	Description	Values/Source	Unit
*r*	Bacteria intrinsic growth rate	Estimated	week$^{-1}$
*K*	Bacteria carrying capacity (maximum sustainable concentration in the water reservoir)	Fixed ($10^6$) [23]	cells liter$^{-1}$
*H*	Half-saturation pathogen density	Fixed ($10^{5.65}$) [23]	cells liter$^{-1}$
$\eta$	Maximum rate of infection	Estimated	week$^{-1}$
$\xi$	Bacteria shed rate	Estimated	cells liter$^{-1}$week$^{-1}$person$^{-1}$
$\mu$	Cholera-induced death rate	Fixed (0.105) [35]	week$^{-1}$
*c*	Minimum Infectious Dose	Fixed ($10^6$) [23]	cells liter$^{-1}$
$\gamma$	Recovery rate	Estimated	week$^{-1}$

We modelled the disease incidence rate when $B \ge c$ using the type-II Holling function [19](see Eq. 2), where $\eta$ is the maximum infection rate and *H* is the half-saturation constant, which specifies the concentration of bacteria that gives half the maximum infection rate. In Eq. (1), $\mu$ is the cholera-induced death rate, $\gamma$ is the recovery rate, and $\xi$ is the rate at which infected individuals shed *V. cholerae* into the water reservoir. We assume that the bacteria concentration grows in the reservoirs with an intrinsic growth rate *r* and a carrying capacity *K*. See Table 2 for a detailed description of the model parameters and their units. The model’s evolution over time is illustarted in Appendix Fig. 14.

#### Basic reproduction number

In a cholera outbreak, to calculate the basic reproduction number of our model, we consider the disease-free equilibrium, where there are no infected individuals in the population. This is possible when the level of bacteria concentration in the reservoir is below the minimum infection dose, when the reservoir’s maximum carrying capacity of the bacteria is lower than this threshold, or when both conditions are met. We assume an initial susceptible population, $S(0) = S_0> 0$, and the initial bacteria concentration, $B^*> 0$, in the reservoir is enough to cause an outbreak. Since $B^*$ is required to trigger the spread of cholera, we impose that the $B^* \ge c$ (see Eq. (2)). Using the next generation matrix approach [8, 53], we compute the basic reproduction number of our model as (see derivation in Appendix 2)

3 $$\begin{aligned} \mathcal {R}_0 = \mathcal {R}_0^{HE} \times \mathcal {R}_0^{B}. \end{aligned}$$

with

$$\begin{aligned} \mathcal {R}_0^B =& \frac{HK \xi }{r(2B^* - K)(B^* - c + H)^2} \quad \\&\text{ and } \mathcal {R}_0^{HE} = \frac{\eta S_0}{ (\mu + \gamma )}, \end{aligned}$$

where $\mathcal {R}^{HE}$ represents the cholera reproductive number in the human population with $\frac{1}{(\mu + \gamma )}$ being the median time spent in the infected compartment and $\eta S_0$ is the portion of the initial susceptibles that moves to the infected compartment, while $\mathcal {R}_0^B$ indicates the contribution of bacteria to the infectivity of cholera in human populations. Finally, $\mathcal {R}_0$, is the basic reproduction number.

### Sensitivity analysis

Sensitivity analysis was performed to determine the impact of the parameters on the basic reproduction number (Eq, (3)). In addition, a sensitivity analysis was performed on the model (Eq (1)) infection peak time and size. We used the Partial Rank Correlation Coefficient (PRCC) method [3, 27, 48].

**Fig. 2:**
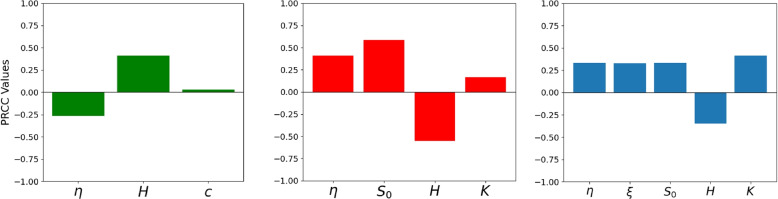
Sensitivity analysis on epidemic peak time, peak size and the basic reproduction number. This depicts the PRCC sensitivity analysis values of **Left panel**: the epidemic peak time. **Middle panel**: the epidemic peak size. **Right panel**: the basic reproduction number. Positive (negative) values of PRCC indicate a positive (negative) correlation between the quantities and the corresponding model parameter, while the magnitude reflects the measure of sensitivity

The ranges of values for the parameters used in the sensitivity analysis are obtained from the posterior distribution table from the STAN output by taking the lowest and highest across the countries except for *H*, *K*, and *c* [24]. Furthermore, $B^*$ was fixed, and the range values of *H*, *c*, and *K* as used in [23] are also used (see Table 3). For the sensitivity analysis, only parameters that showed a meaningful effect on outbreak dynamics were considered, as shown in Fig. 2.

**Table 3:** Parameter ranges used in the PRCC sensitivity analysis. Bounds reflect the minimum and maximum values observed across all countries. Values for $H$, $K$, and $c$ are taken from [24]. A uniform distribution (U) was assumed for each parameter

Parameter	Lower bound	Upper bound	Distribution
$\xi$	0	700	U
$\eta$	0.1	1	U
$S_0$	1	36058	U
$H$	$10^{6}$	$10^{8}$	U
$\gamma$	0.15	5	U
$r$	2.1	100.1	U
$c$	$10^5$	$10^7$	U
$K$	$10^5$	$10^7$	U

The PRCC values were computed for every parameter using 10, 000 samples generated by Latin Hypercube Sampling (LHS). The findings revealed how much each parameter affects $\mathcal {R}_0$, the epidemic peak time, and peak size. In addition, a bivariate distribution was considered for parameters $B^*$ and *K* to ensure the inequality $2B^*> K$, using the conditional sampling of $B^* \sim \mathcal {U}(K/2 + \varepsilon ,\ K)$, with $\epsilon>0$ to make $\mathcal {R}_0$ valid. Moreover, samples were accepted when the parameters $K>c$ and bivariate normal distributions were used to jointly sample the parameters, with mean vector $[5 \times 10^5,\ 5 \times 10^6]$ and covariance matrix

$$\begin{aligned} \left[ \begin{array}{ll} 1 \times 10^{10} & 3 \times 10^8 \\ 3 \times 10^8 & 1 \times 10^{12} \end{array}\right] . \end{aligned}$$

to ensure the bacteria’s maximum capacity threshold exceeds the minimum infection dose for an outbreak. A non-negative PRCC value indicates that at any slight increment in the parameter, the corresponding outcome also increases; whereas a non-positive value means that an increase would lead to a decrease in the outcome.

Sensitivity analysis emphasizes the varying impact of factors on the reproduction number $(\mathcal {R}_0)$. Factors such as the susceptible population ($S_0$), the maximum infection rate $(\eta )$, the human shedding rate ($\xi$) and the maximum carrying capacity (K) demonstrate strong positive relationships with $(\mathcal {R}_0)$, indicating that increases in these factors notably increase the potential for spread of the disease. In contrast, the half-saturation bacteria density (*H*) exhibits negative associations, implying that higher values of these factors lead to a decrease. The outcome of the sensitivity analysis was illustrated in Fig. 2

Various model parameters affect the epidemic peak time and peak size. To evaluate their relative importance, a sensitivity analysis was performed using PRCC. The result indicates that the maximum infection rate $(\eta )$, initial susceptibles and carrying capacity (*K*) positivity affect the epidemic peak size and the negative effect of the density of half-saturation bacteria, while the half-saturation bacteria density (*H*) had a more positive influence on the timing of the epidemic peak.

Overall, these results underscore the significance of managing the maximum infection rate, initial susceptibles, human shedding rates, and carrying capacity to effectively control and reduce disease transmission, as these play an essential role in the transmission of the infection.

### Data

The data used was meticulously gathered from the Global Cholera and Acute Watery Diarrhoea (AWD) Dashboard [38], which provides updates on the cholera cases to the WHO between 2022 and 2024 in regions where cholera is prevalent. The primary focus is on African nations that were significantly impacted during this period, including Mozambique, Zambia, and Zimbabwe. These countries have experienced notable cholera outbreaks, making them crucial for studying disease patterns and evaluating the effectiveness of intervention measures.

Additionally, the study incorporates data from other African countries reporting cholera cases during this timeframe, such as Comoros, Cameroon, Somalia, and Sudan. The data set includes weekly records of cases from the countries studied.

### Bayesian inference

The model fitting for Eq. (1) was done using a Bayesian inference framework. Parameter estimation was achieved by fitting the model to the weekly confirmed cholera cases using the No-U-Turn Sampler(NUTS) MCMC algorithm implemented with STAN via RStan package in R-language. The prior distributions across countries are summarized in Appendix Table 4.

The initial conditions for the iSIRB model (Eq. (1)) used for model fitting are summarized in Table 2. Since the fraction of the population that interacts with reservoirs of the cholera pathogen is of concern, initial susceptibles *S* were estimated. Furthermore, the initial infection was estimated to increase variability as the initial infected count can be used instead, but this might not give the true value.

In the STAN model, the likelihood function was defined as:

4 $$\begin{aligned} \textrm{ComfirmedCases}(t) \sim \textrm{NegBin}(\text{ incidence }(t), \phi ) , \end{aligned}$$

$\textrm{NegBin}(\cdot )$ denotes the negative binomial distribution, $\textrm{ComfirmedCases}(t)$ is the total weekly reported cholera cases obtained from the data [38], incidence(*t*) is the weekly incidence of cholera computed using our model Eq. (1) (this is calculated as the number of susceptible individuals transitioning into the infected compartment weekly), and $\phi$ is the dispersal parameter. $\phi$ captures the degree of overdispersion, which occurs when the variance exceeds the mean. While a large $\phi$ implies low overdispersion, a small $\phi$ shows high overdispersion. This allows effective calculation using the NUTS algorithm with 5,000 iterations and four chains.

The model’s validation was confirmed by comparing how well its predictions were fitted to the weekly confirmed cholera cases, with a Gelman–Rubin diagnostic ($\hat{R}$) score close to one. This metric assesses the convergence of the MCMC simulations, ensuring the chains are well mixed and fully represent the posterior distribution. A range of values that suggest satisfactory convergence is between 1 and 1.02.

### Model fitting and parameter estimation

**Fig. 3:**
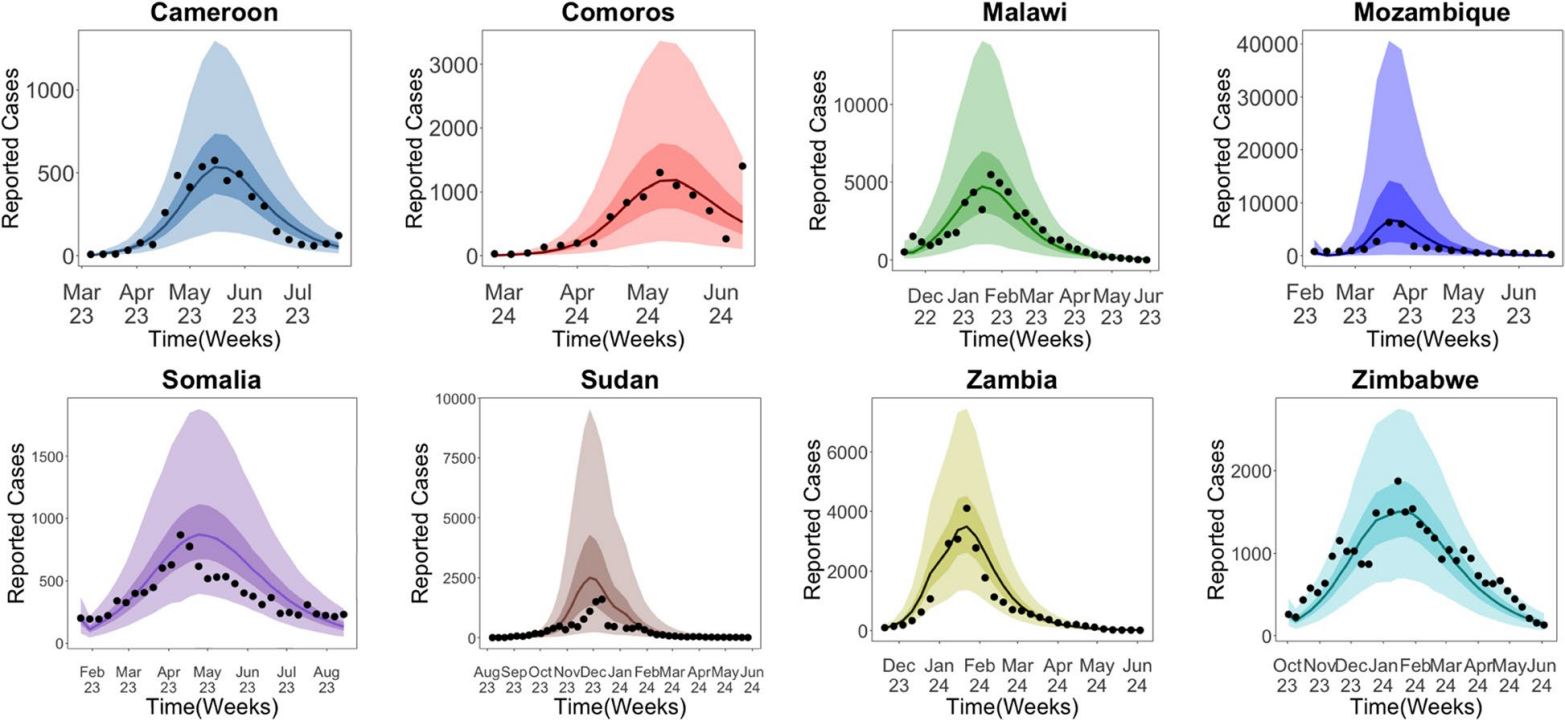
Model fit to weekly reported cholera cases. Black dots represent the weekly reported cholera cases in the countries, while the solid lines are the median predicted cases. The narrower, darker bands represent the 50% credible interval (CrI), and the wider, lighter bands represent the 95% CrI. The period shown corresponds to cholera outbreaks in Africa between 2022 and 2024, with days selected based on the outbreak timeline for each country

Each country’s weekly cases were fitted with STAN. The bacteria carrying capacity *K*, the minimum infection dose *c*, and the cholera-induced death rate $\mu$ were assumed to be consistent in all countries. However, the density of half-saturation bacteria *H* varies from country to country. Figure 3 presents the iSIRB model fits to weekly reported cholera cases across the eight countries.

The rest of the parameters were estimated to ensure the model captures the dynamics of cholera transmission well. Additionally, the initial number of susceptible and infected individuals was estimated, although not considered a model parameter, to give the model a good starting point. The parameter values estimated for each country are summarized in Appendix Tables 5 and 6.

### Statistical analysis

Normality was assessed using the Kolmogorov-Smirnov test [47] on the posterior distribution of the model parameters and the initial values of *S* and *I*. The resulting *p*-values predominantly clustered around zero (see Appendix 5.1), indicating that the normality assumption was not satisfied for most parameters and variables. We also evaluated the homogeneity of variance using Levene’s test (see Appendix Table 10). The results indicated a p-value $>0.005$ for all parameters, confirming a violation of the equal variance assumption.

**Fig. 4:**
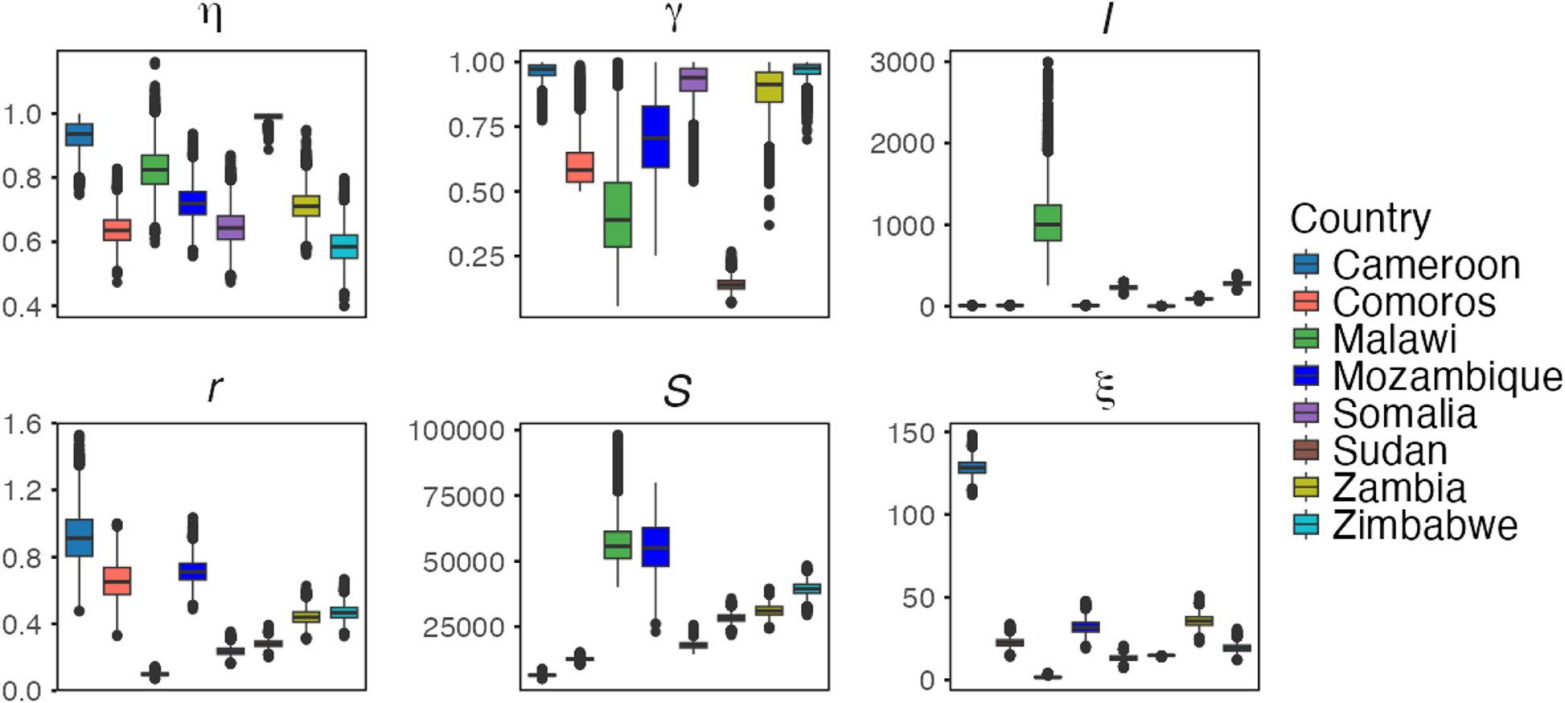
Cross-Country Comparison of Parameter Distributions and Statistical Significance. Box plots illustrate the distribution of parameters across countries. The Kruskal-Wallis test *p*-values assess whether parameter distributions differ significantly. All parameters yield *p*-values below $2 \times 10^{-16}$, demonstrating statistically significant differences in parameter distributions between countries. This highlights variations in cholera outbreak characteristics across Cameroon, Comoros, Malawi, Mozambique, Somalia, Sudan, Zambia, and Zimbabwe, emphasizing regional disparities in infection dynamics across Africa

Given that both conditions were unmet, we employed a non-parametric test (Kruskal-Wallis [25]) to compare parameter distributions across African countries. The distribution of the estimated parameters across the considered countries is illustrated in Fig. 4

### Hierarchical clustering

Hierarchical clustering was employed to group countries exhibiting similar cholera dynamics based on median estimated parameters (see Appendix Table 7) and confirmed cholera cases.

**Fig. 5:**
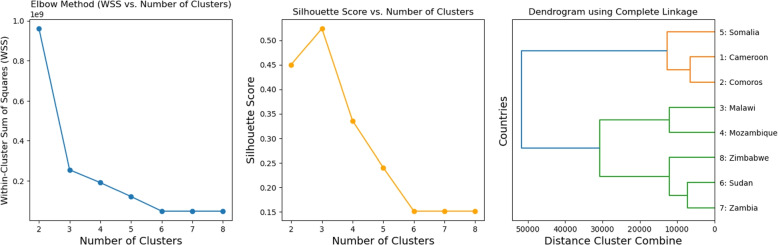
Clustering Analysis: **Left panel: The Elbow Method**—This method determines the optimal number of clusters by computing the Within-Cluster Sum of Squares for different cluster counts. The optimal value is identified visually at the elbow of the plot, which is three in this case. **Center panel: Silhouette Score Plot**—This plot evaluates the optimal number of clusters by computing and plotting the silhouette score against (number of clusters). The highest silhouette score corresponds to the optimal clustering, which in this case is three, further confirming the hierarchical clustering result. **Right panel:** Hierarchical clustering dendrogram obtained using the Complete Linkage method

The dendrogram (See Fig. 5) revealed three distinct clusters. Cluster 1 includes Somalia, Cameroon and the Comoros; Cluster 2 includes Malawi and Mozambique; Cluster 3 includes Zimbabwe, Sudan, and Zambia. Cluster quality was evaluated using the silhouette score, which produced a value of 0.52, indicating a moderate level of separation between groups. To further investigate these clusters and identify significant parameters for targeted health responses, additional statistical analyses were conducted. The normality of parameter and variable distributions across clusters was assessed (See Appendix Table 9 for the parameters and Appendix 5.2), and homogeneity of variance was evaluated for Clusters 1 and 3 due to their sample (See Appendix Table 10). In Cluster 2, which contains only two countries, the normality test used for other clusters was inapplicable. Instead, a heuristic rule-of-thumb method was employed, revealing that normality was not satisfied for any parameter or variable.

Given these findings, statistical tests were applied selectively. For parameters and variables meeting parametric assumptions, ANOVA was used; otherwise, the Kruskal-Wallis test was employed. The results indicate that most variables do not exhibit significant differences ($p> 0.05$). However, the variable *S* (initial susceptible population), which demonstrated normality in both Cluster 1 and Cluster 3, did show a significant difference in medians across clusters, as indicated by the One-Way ANOVA test ($p = 0.0121$; Appendix Tables 11 and 12).

## Results

### Maximum infection rate $(\eta )$

**Fig. 6:**
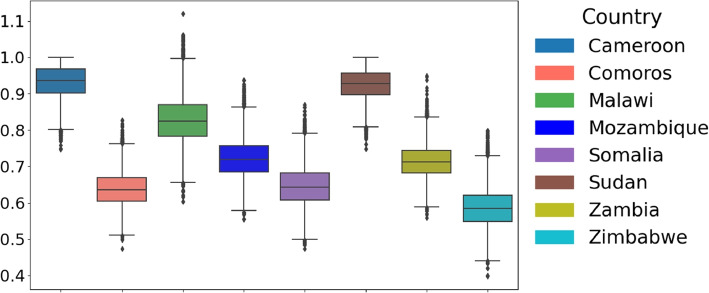
Distribution of parameter $\eta$ across countries: Boxplot showing the distribution of parameter $\eta$ across the considered eight countries: Cameroon, Comoros, Malawi, Mozambique, Somalia, Sudan, Zambia, and Zimbabwe. The central line in each box represents the median, while the box indicates the interquartile range (IQR)

Among the countries considered in this study, the highest maximum infection median estimate was found in Sudan with a median estimate of 0.99, indicating a severe outbreak with a high transmission rate. The lowest value was that of Zimbabwe, 0.59, which indicated a less severe outbreak compared to the other studied countries. Other noteworthy values were those of Cameroon, 0.93; Comoros, 0.64; Mozambique, 0.72; Somalia, 0.65; Sudan, 0.93; and Zambia, 0.71. These maximum infection rates differ from country to country (see Fig. 6). They are accounted for by differences in the intensity of outbreaks, possibly due to variations in public health infrastructure, intervention strategies, and population behaviour.

### Recovery rate ($\gamma$)

**Fig. 7:**
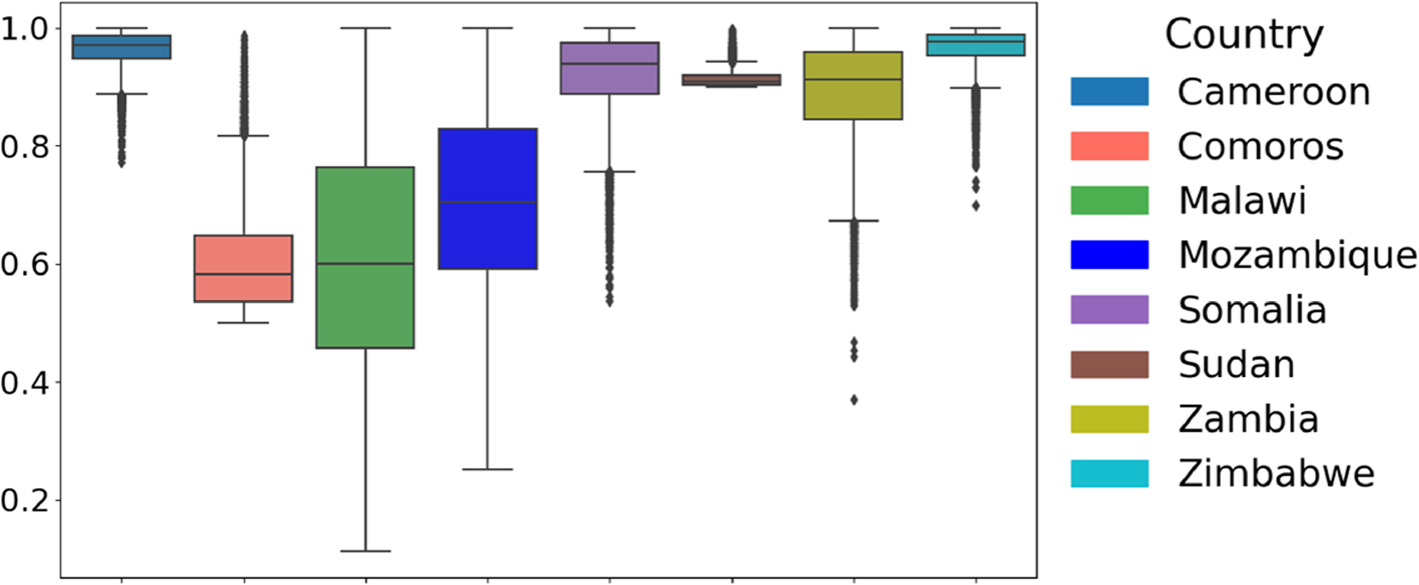
Distribution of parameter $\gamma$ across countries: Boxplot showing the distribution of parameter $\gamma$ across the considered eight countries: Cameroon, Comoros, Malawi, Mozambique, Somalia, Sudan, Zambia, and Zimbabwe. The central line in each box represents the median, while the box indicates the interquartile range (IQR)

The distribution of recovery rate across countries is shown in Fig. 7. In Comoros, the recovery rate ranged between 0.50 and 0.65, with a median of 0.60, while in Mozambique, it ranged from as low as 0.41 to as high as 0.98, with a median of 0.71. These medians indicate that health outcomes differ and might characterize varying levels of healthcare quality or accessibility. In contrast, Zimbabwe exhibited an overall median recovery rate of 0.97, which ranged from 0.88 to 1.00. This could indicate that health responses have been consistent or that the severity of the disease was less prominently manifested. Other countries that had a good median recovery rate include Cameroon with 0.96, Sudan with 0.91, Somalia with 0.92, and Zambia with 0.89. In addition, Malawi has a recovery rate of 0.41, potentially linked to inadequate sanitation and insufficient access to safe drinking water.

### Bacterial growth rate (r)

**Fig. 8:**
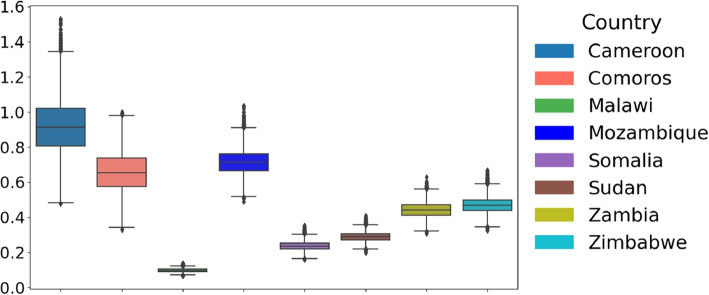
Distribution of parameter *r* across countries: Boxplot showing the distribution of parameter *r* across eight countries: Cameroon, Comoros, Malawi, Mozambique, Somalia, Sudan, Zambia, and Zimbabwe. The central line in each box represents the median, while the box indicates the interquartile range (IQR)

Cameroon had a rate of 0.92 (95% CrI 0.64 - 1.24), suggesting higher bacterial growth than other regions. In contrast, Mozambique recorded a median rate of 0.72, Comoros (0.66) and Malawi (0.10), Somalia (0.24), Sudan (0.29), and Zimbabwe (0.47), and Zambia at (0.44) for the bacteria growth rates. These differences in the bacteria’s growth rates (see Fig. 8) may imply differing environmental factors and strain characteristics influencing the outbreaks.

### Initial susceptible population ($S_0$)

**Fig. 9:**
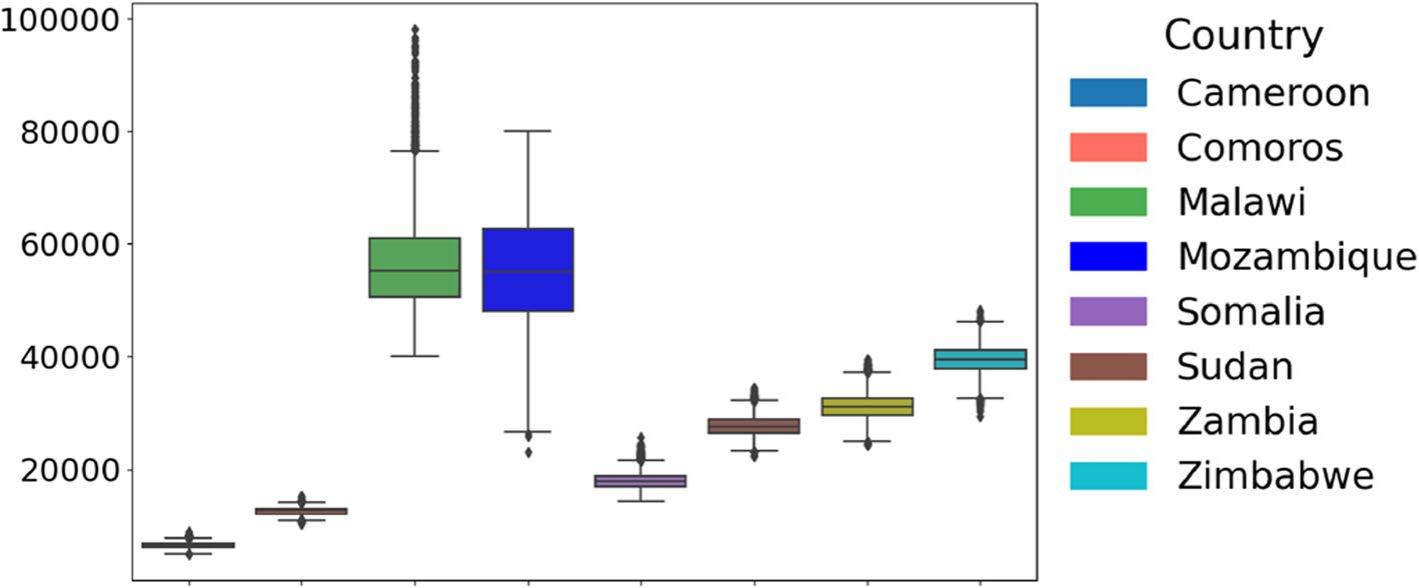
Distribution of parameter *S* across countries: Boxplot showing the distribution of parameter *S* across the considered eight countries: Cameroon, Comoros, Malawi, Mozambique, Somalia, Sudan, Zambia, and Zimbabwe. The central line in each box represents the median, while the box indicates the interquartile range (IQR)

Regarding the initial susceptible population denoted as $S_0$, there were significant differences in the estimates among the considered countries, reflecting differences in the portion of the population exposed to the bacteria (see Fig. 9). Malawi’s estimate was the highest, with a median estimate of 56, 280, followed closely by Mozambique at 55, 546. The next highest estimate was Zimbabwe and Zambia, with 39, 425 and 31, 178, respectively. Other Countries have lower estimates; including Sudan (27, 704), Comoros (12, 564), Cameroon (6, 438), and Somalia (17, 963).

### The initial infected population ($I_0$)

**Fig. 10:**
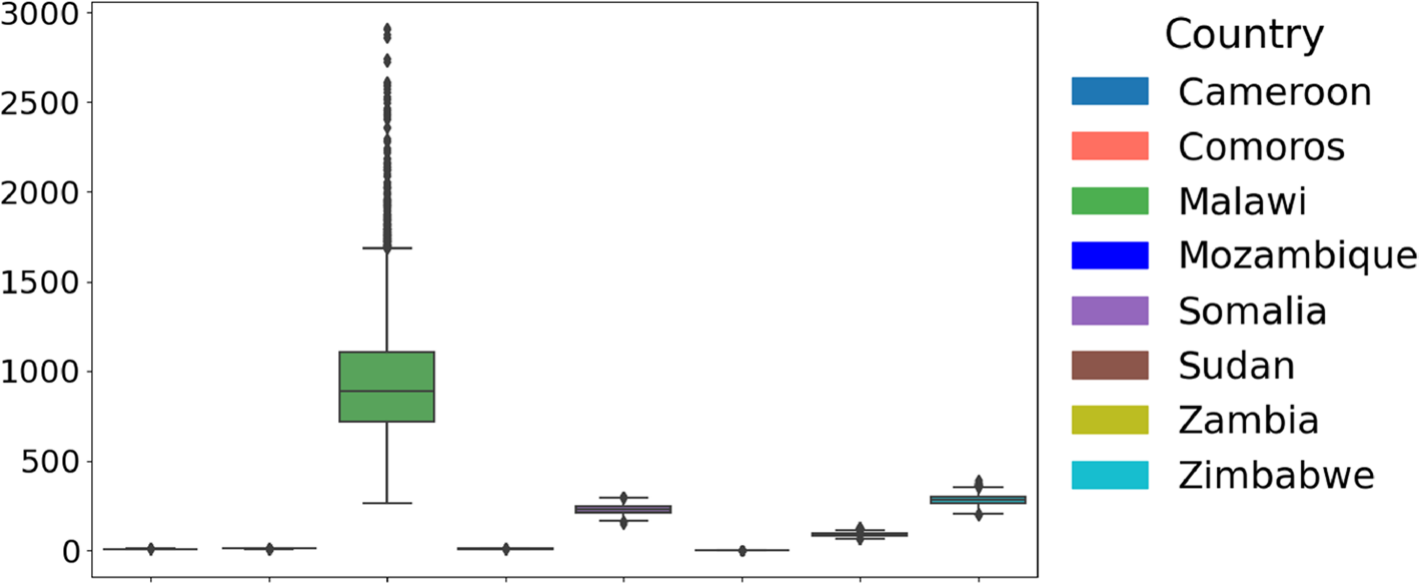
Distribution of parameter *I* across countries. The Boxplot shows the distribution of parameter $I_0$ across the considered eight countries: Cameroon, Comoros, Malawi, Mozambique, Somalia, Sudan, Zambia, and Zimbabwe. The central line in each box represents the median, while the box indicates the interquartile range (IQR)

The initial distribution of the infected population between countries is shown in Fig. 10. In Malawi, the initial infected were the highest, with a median of 1054 ($95\%$ CrI 476 - 1657). Zambia and Somalia also had median initial infected estimates of 90 and 230, respectively, indicating spread but with moderate variability. On the other hand, Sudan had a median of 1, suggesting a minimal initial cholera infection at the start of the outbreak. Countries like Mozambique(8) and Zimbabwe(281), along with Comoros and Cameroon, have a median of 9 and 7.

### The shedding rate ($\xi$)

The shedding rate ($\xi$) reflects the infected individuals’ contribution to cholera transmission. Higher shedding rates indicate poor fecal disposal, limited access to sanitation, and improper care of infected individuals. Cameroon exhibited the highest shedding rate, with a median of 128.32, indicative of intense bacterial spread by the infected, which was likely driven by low sanitation, high transmission rates and environmental conditions. Zambia followed with a median of 35.77. Zimbabwe has a median shedding rate of 19.36, Mozambique (32.03), Somalia (13.27), Comoros (22.56) and Sudan (19.36).

**Fig. 11:**
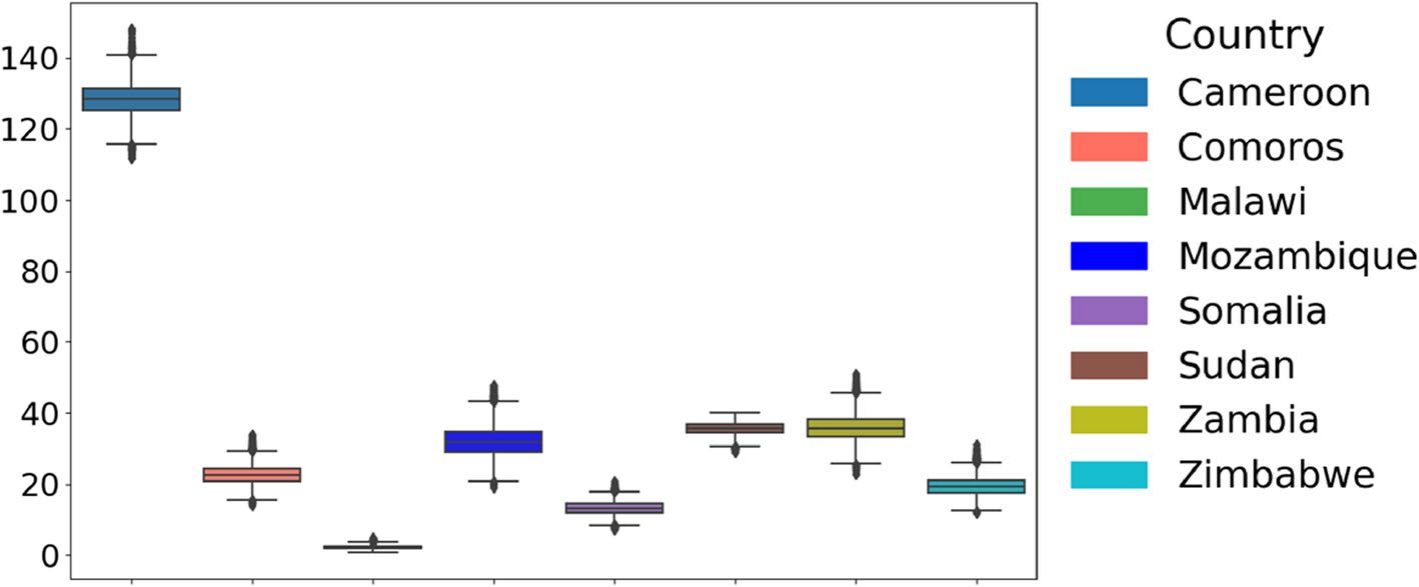
Distribution of parameter $\xi$ across countries: Boxplot showing the distribution of parameter $\xi$ across the considered eight countries: Cameroon, Comoros, Malawi, Mozambique, Somalia, Sudan, Zambia, and Zimbabwe. The central line in each box represents the median, while the box indicates the interquartile range (IQR)

In Malawi, the shedding rate was the lowest among the countries, with a median of 1.75. These differences in values across regions (see Fig. 11) highlight the role of localized interventions, environmental factors, and access to resources in managing cholera transmission and control efforts.

### Basic reproduction number ($\mathcal {R}_0$)

**Fig. 12:**
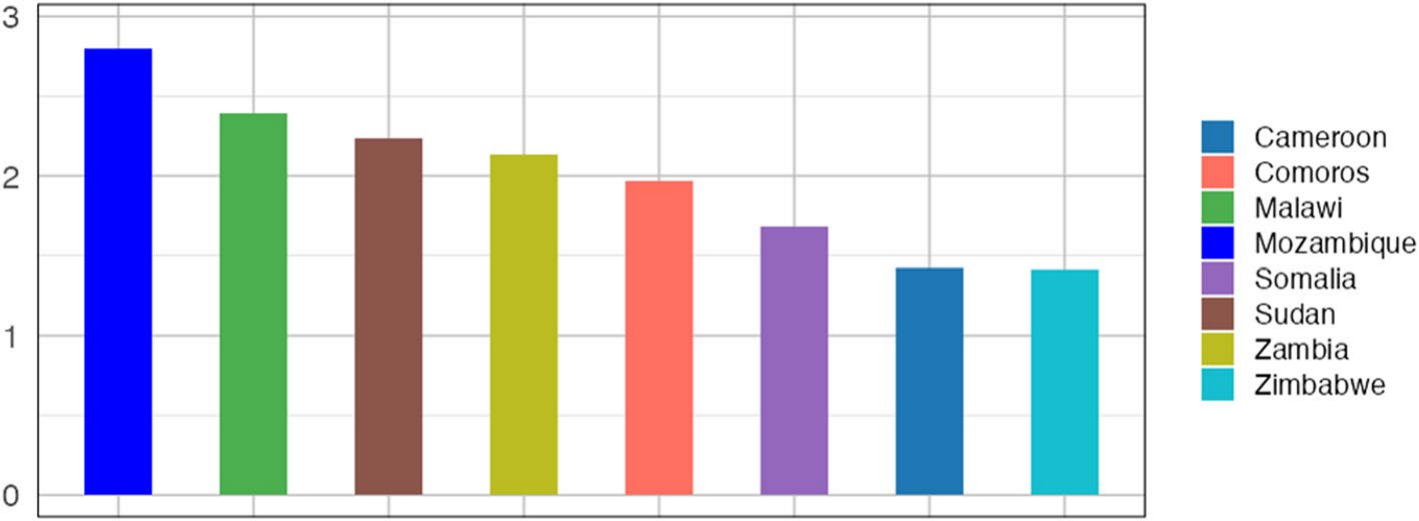
Basic reproduction number: The basic reproduction number of cholera in each country for the specific epidemic considered in the country (shown in Fig. 3)

We computed the basic reproduction number $(\mathcal {R}_0)$ for each country shown in Fig. 12. The country with the highest $\mathcal {R}_0$ was Mozambique, with an $\mathcal {R}_0$ of 2.80, hence a high transmission potential warranting any urgent control measure. The lowest $\mathcal {R}_0$ was for Zimbabwe at 1.41, with low transmission risk but still above the threshold for epidemic potential. Other notable $\mathcal {R}_0$ values were for Cameroon, 1.42; Comoros, 1.97; Malawi, 2.39; Sudan 2.21; Somalia, 1.78; and Zambia, 2.13. These values underscore the differing levels of outbreak control required in each country.

## Discussion

In cholera-endemic regions in Africa, the infection continues to pose a threat to public health due to persistent socioeconomic and environmental vulnerabilities. Cholera transmission is exacerbated by limited access to clean water, poor sanitation infrastructure and recurrent natural disasters. Despite efforts to reduce its impact, many countries struggle to address these underlying factors, underscoring the importance of sustainable solutions to reduce the burden of cholera. These socioeconomic factors affect the model parameters, such as the bacteria concentration in water (via open defecation and poor hygiene practices), maximum infection rate (via low EPI Drinking Water Scores), and recovery rate (via healthcare access), all of which are mechanistically incorporated into the model.

**Fig. 13:**
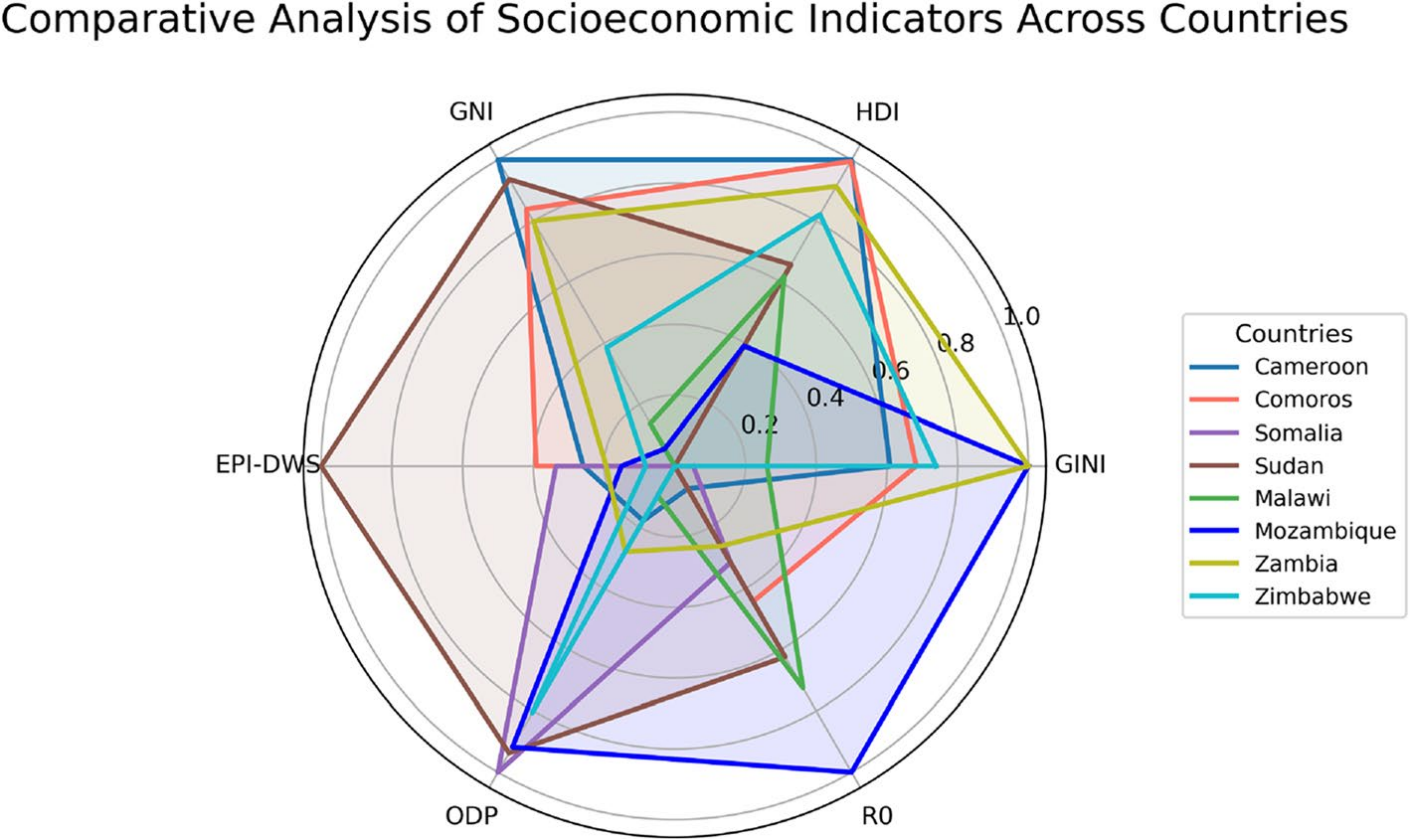
Radar chart comparing socioeconomic indicators and the basic reproduction number $\mathcal {R}_0$ across the considered eight countries. The chart visualizes normalized values of six variables: GINI index, Human Development Index (HDI), Gross National Income (GNI), EPI Drinking Water Score (EPI-DWS), and Open Defecation Percentage (ODP) (see Appendix Table 8), along with the basic reproduction number $\mathcal {R}_0$

This study provides insight into the dynamics of cholera transmission in eight African countries during the 2022-2024 outbreaks, highlighting differences in transmission potential, with basic reproduction number $\mathcal {R}_0$ ranging from 1.41 in Zimbabwe to 2.80 in Mozambique (see Fig. 12). Key factors affecting the outbreaks include the maximum infection rate, human shedding rate, and initial susceptible population, while the recovery rate across these countries varies, with Malawi experiencing the lowest and Zimbabwe exhibiting the highest. This relationship is further shown in a Rader chart in Fig. 13 and Appendix Fig. 15.

The results demonstrate connections between socioeconomic indicators and cholera transmission across the considered counties (Fig. 13 and Appendix Fig. 15). Malawi, with the highest number of initial susceptible populations, has one of the lowest EPI Drinking Water Score (EPI-DWS) and had a high $\mathcal {R}_0$ of 2.39, aligning with its several outbreaks. Likewise, Mozambique had a high $\mathcal {R}_0$ of 2.80, has a low EPI-DWS, and GNI with a high open defecation percentage, emphasizing the link between economic vulnerability, infrastructure and cholera severity. In contrast, despite having a relatively better EPI-DWS and GNI, Cameroon, Somalia, and Sudan exhibit a high $\mathcal {R}_0$ attribute to their high open defecation percentage, underscoring the role of sanitation in driving cholera transmission. Furthermore, Comoros and Zambia, despite having moderate GNI, HDI and GINI but low ODP, still display high $\mathcal {R}_0$ of 1.97 and 2.13, respectively, which is attributed to significant human shedding of the bacteria back to the environment, which is not mitigated through infrastructure. The low basic reproduction number in Cameroon and Zimbabwe can be attributed to the under-reporting of cases.

To further analyze these patterns, hierarchical clustering was used to group countries based on their socioeconomic indicators and cholera metric (the estimated parameters and variables), revealing distinct clusters of countries sharing similar cholera dynamics, offering region-wise risk factors. The clustering identified three groups: Natural disaster-affected countries (Malawi and Mozambique), Economic and Infrastructure Challenged countries (Sudan, Zimbabwe and Zambia) and countries with chronic sanitation issues (Somalia, Cameroon and Comoros).

Natural disaster-affected countries (e.g., cyclones, floods), exhibit high $\mathcal {R}_0$ values and have a low EPI-DWS, highlighting their vulnerability to floods and cyclones that disrupt sanitation systems. For example, Tropical Storm Ana in Malawi in 2022 and Cyclone Freddy in Mozambique in 2023 both destroyed Water Sanitation and Hygiene infrastructure leading to the limited supply of clean water thus, increasing the risk of cholera [44, 50].

Economic and infrastructure-challenged countries have a basic reproduction number that is above 2.0, attributed to systemic socioeconomic and infrastructural challenges. In Sudan, the armed conflict has severely disrupted the health services, damaged infrastructure, and displaced people, resulting in a multi-state cholera outbreak [11]. Zimbabwe and Zambia face similar challenges, having weak infrastructure, economic instability and poor access to safe water and sanitation [17, 51].

Additionally, countries with chronic sanitation experience persistent cholera transmission mainly because of low sanitation infrastructure and hygiene practices. Somalia faces recurring outbreaks with an open defecation rate of $34\%$, $80\%$ Somali families lack a hand-washing facility and about $28\%$ lack a functional sanitation facility [52]. Similarly, in Cameroon, the related risk factor encouraging the spread of cholera is due to the county’s open defecation, inadequate safe water and poor food handling[33]. Also in Comoros, at the center of the outbreak is the Island of Anjouan where about $60\%$ of cholera-induced deaths occur. The island’s primary water supply, the river contaminated by waste, has become a major transmission pathway [43].

Despite these results, this study has certain limitations that should be given thought. The first is the reliance on data from only eight African countries for parameter estimation and hierarchical clustering, which restricts the generalization of the findings to all cholera-affected countries in Africa. This limitation is due to little of no availability of data in other countries. Though these countries have different major outbreak drivers, including natural disasters, economic and infrastructural challenges, and chronic sanitation, not considering other African nations restricts the ability to fully understand the dynamics of the infection across the continent. The inclusion of all cholera-affected countries will provide a broader understanding of the factors driving cholera transmission. Another limitation is the quality and consistency of the reported cholera-confirmed cases dataset used in the data fitting process. The study uses publicly available data from the WHO [38], which may be affected by under-reporting, delays, or disparity like in conflict-affected regions (Sudan and Somalia), where the health system is disrupted. In these places, the collection of data might not reflect the actual value of cholera cases.

## Conclusion and recommendations

This study explored model-based and data-driven understanding of cholera transmission dynamics in African countries with persistent outbreaks between 2022 and 2024. Bayesian Parameter estimation on important model parameters was carried out using STAN. Through this, we identified parameters and epidemiological factors affecting cholera transmission across the considered countries. This sheds light on factors driving frequent cholera outbreaks, such as human bacterial shedding rate, recovery rate, maximum infection rate and initial susceptibles likely to be exposed to *Vibrio cholerae* in eight African countries. Sensitivity analysis gives insight into how small changes in these parameters can affect outbreak peak size, timing and the basic reproduction number. In addition, hierarchical clustering helped stratify the countries, revealing three distinct groups. Within these groups, the study found that the countries prone to natural disasters (Malawi and Mozambique) tend to have high $\mathcal {R}_0$ and bacteria shedding rates. Economically and infrastructurally challenged countries (Sudan, Zambia and Zimbabwe) are impacted by poor recovery rates and high initial susceptible populations, indicating a need for improved health care and access to clean water. Meanwhile, in countries facing chronic sanitation issues (Comoros, Cameroon and Somalia), the infection persists due to the high transmission rate and bacterial shedding, pointing to the importance of addressing sanitation deficiencies, eliminating open defecation and promoting good hygiene practices in reducing the infection. Though this study provides good insight into cholera transmission, its dependence on eight countries’ cholera data and potential inconsistencies call attention to the need for the inclusion of more African cholera-affected countries’ datasets and improved data quality in future research.

Based on the results of this study, several recommendations can be suggested to reduce the adverse cholera transmission in these countries. Countries like Malawi and Mozambique should emphasize the strengthening of the disaster response system by incorporating the WASH framework into their disaster preparedness plan before and after floods and cyclones to reduce their high $\mathcal {R}_0$, human bacteria shedding rate and the maximum infection rate. For countries with economic and infrastructure challenges, the focus should be on rebuilding and enhancing public health infrastructure to improve the recovery rate and reduce initial susceptibles. Also, strengthening waste management systems and improving water quality can address the high $\mathcal {R}_0$ values and persistent outbreaks in these countries. Lastly, in countries with chronic sanitation issues, intervention should be on addressing sanitation deficiencies to reduce the transmission rate and reduce bacteria shedding. In terms of their socioeconomic indicators, the focus should be on eliminating open defecation and improving waste disposal practices. This can be done with hygiene education campaigns to promote behavioural changes and reduce transmission. Overall, implementing measures tailored to the specific needs of each region will reduce cholera cases in such regions and improve public health outcomes.

## Supplementary Information

Supplementary Material 1.

## Figures and Tables

**Fig. 14:**
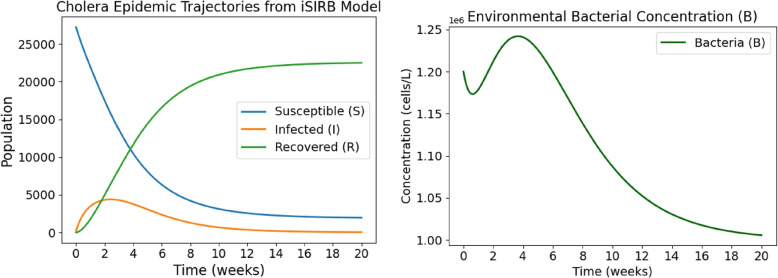
Simulated cholera epidemic dynamics using the iSIRB model. **Left Panel**: shows the simulated trajectories of the susceptible (S), infected (I), and recovered (R) populations. **Right Panel**: Depicts the environmental bacterial concentration (*B*) over time. The model incorporates logistic bacterial growth and pathogen shedding by infected individuals. Parameter values and initial conditions are averaged across eight African countries with observed cholera outbreaks between 2022 and 2024. The simulations reflect average epidemic behaviour based on reported regional conditions

**Fig. 15:**
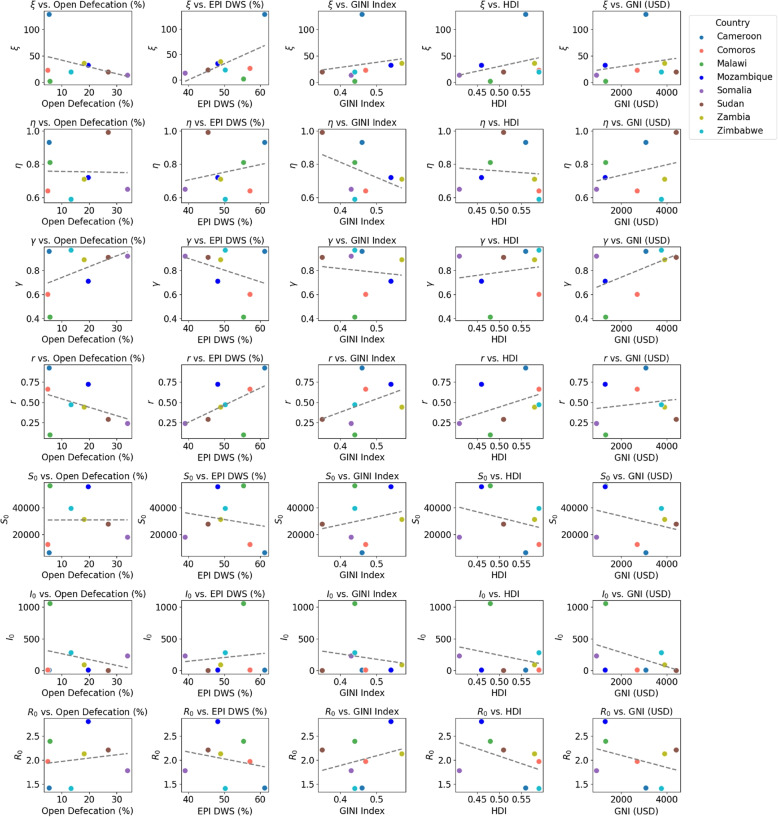
Relationship between model parameters and socioeconomic indicators. Each panel shows a scatter plot with a linear regression line, comparing a calibrated epidemiological parameter to a country-level factor across eight African countries

**Fig. 16:**
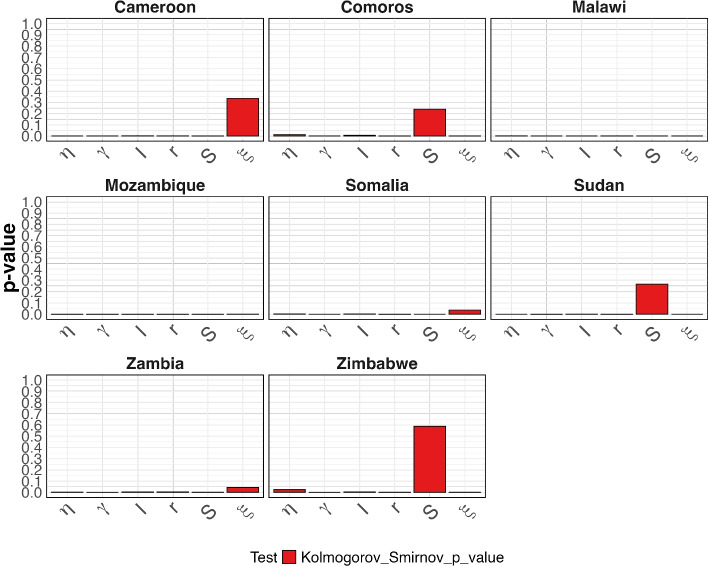
Normality Test Results: Bar plot showing the *p*-values from Kolmogorov-Smirnov normality tests for different parameters across countries. Each bar represents the p-value of a specific test for a given parameter, grouped by country. A p-value above 0.05 generally indicates that the data does not deviate significantly from normality

**Fig. 17:**
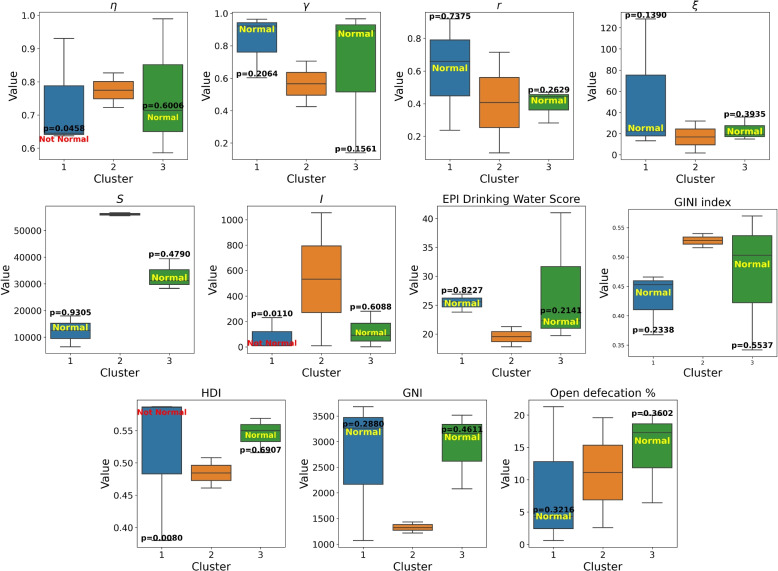
Box Plots of Parameters with Cluster-wise Normality Assessment and *P*-Values. The figure displays box plots for the analyzed parameters across three clusters. Each box plot includes annotations for Shapiro-Wilk test *p*-values and normality status. Parameters marked as *Normal* (green/yellow) passed the normality test ($p> 0.05$), while *Not Normal* (red) indicates deviations from normality ($p \le 0.05$). The values are distributed across clusters to assess inter-cluster variability

## Data Availability

The cholera data used for our study is publicly available and can be obtained from the WHO Cholera and AWD dashboard (https://who-global-cholera-and-awd-dashboard-1-who.hub.arcgis.com/).

## Appendix

### Disease-free equilibriumpoint

For the disease-free equilibrium, we consider a situation where $I = 0$:

$$\begin{aligned} \left\{ \begin{array}{l} 0 = -\alpha (B) S \\ 0 = \alpha (B) S - (\gamma + \mu ) I = 0 \implies I = 0 \\ 0 = \xi I + r B \left( 1 - \frac{B}{K}\right) = 0 \implies B = 0 \text { or } B = K \end{array}\right. \end{aligned}$$

This leads to the disease-free equilibrium points:

$$\begin{aligned} E_1= (S, I, B) = (S_0, 0, 0) \end{aligned}$$

$$\begin{aligned} E_2 =(S, I, B) = (S_0, 0, K) \quad \text {for } K < c \end{aligned}$$

The condition $K \le c$ is necessary for the absence of cholera.

The final disease-free equilibrium point occurs when the environmental bacterial level is low enough not to trigger cholera infections within the human population. The level will be donated by $B_0$

$$\begin{aligned} E_3 = (S, I, B) = (S_0, 0, B^*) \quad \text {for } B^* < c \end{aligned}$$

This is true because these regions have bacteria during the inter-epidemic period, which is not enough to cause an outbreak unless influenced by outside factors.

### Basic reproduction number

Define the transmission matrix as the introduction of infection in the *I* and *B* compartments, $\mathcal {F}$ given as

A1 $$\begin{aligned} \mathcal {F} := \left[ \begin{array}{c} \dfrac{\eta (B-c)}{(B-c)+H} S \\ 0 \end{array}\right] . \end{aligned}$$

While the transition matrix is given as

A2 $$\begin{aligned} \mathcal {V} := \left[ \begin{array}{l} \mu I + \gamma I \\ -r B \left( 1 - \frac{B }{K} \right) - \xi I \end{array}\right] , \end{aligned}$$

The Jacobian matrix of the matrices 5 and 6 at the disease-free equilibrium point $(E_3)$ are

A3 $$\begin{aligned} F= \left[ \begin{array}{cc} 0 & \dfrac{\eta S_0 H }{\left( B_0-c+H\right) ^{2}} \\ 0 & 0 \end{array} \right] , \quad V= \left[ \begin{array}{cc} \mu + \gamma & 0 \\ - \xi & -r \left( 1 - 2\frac{B_0 }{K} \right) \end{array} \right] . \end{aligned}$$

A4 $$\begin{aligned} F V^{-1} = \left[ \begin{array}{cc} - \frac{\eta S_0 H \xi K}{ r\left( B^*-c+H\right) ^{2} \left( \gamma +\mu \right) \left( K-2 B_{0}\right) } & -\dfrac{\eta S_0 H K}{\left( B^*-c+H\right) ^{2} r( K-2 B^*)} \\ 0 & 0 \end{array} \right] , \end{aligned}$$

The basic reproduction number is the spectral radius of the $FV^{-1}$, the largest positive eigenvalues. This

A5 $$\begin{aligned} \mathcal {R}_0 = \dfrac{\eta \xi S_0 H K}{ r\left( \mu +\gamma \right) ( 2 B^*- K) \left( B^* -c + H\right) ^{2}}. \end{aligned}$$

is the basic reproduction number if only $2 B^{*}>K$ and $B^* \ne H-c$.

Another basic reproduction occurs when the bacteria are absent in the region (i.e $B^* = 0$). That is, the disease-free equilibrium is $(S_0,0,0)$. Here, the basic reproduction number is

A6 $$\begin{aligned} \mathcal {R}_0 = \dfrac{\eta \xi S_0 H }{ r\left( \mu +\gamma \right) \left( H -c\right) ^{2}}. \end{aligned}$$

This basic reproduction number is valid if the initial concentration of bacteria is greater than its maximum carrying capacity and $H\ne c$. This assumption is realistic, as at the start of an outbreak, the initial concentration of the bacteria is greater than its maximum capacity(*K*) $\left[ B^* \ge K \right]$. Thus, from the dynamics of the logistic growth model, the initial concentration will decrease to or past the maximum carrying capacity over time.

When $$B^*>K$$

$$\begin{aligned} \left( 1 - \frac{B}{K}\right) <0 \end{aligned}$$

thus, the logistic growth model can be rewritten as

$$\begin{aligned} rB \left( 1 - \frac{B}{K}\right) <0 \end{aligned}$$

The population is unsustainable here, and natural forces drive it towards or below the maximum capacity.

When $B^* = K$, human sheddingkeeps the bacteria level above the maximum carrying capacity up to a level required for an outbreak since it is non-negative.
