# Supplementary material for: Understanding cholera dynamics in African countries with persistent outbreaks: a mathematical modeling approach
Source: BMC Public Health. 2025 Oct 8;25:3395. doi: 10.1186/s12889-025-24507-0 (PMC12506165; doi:10.1186/s12889-025-24507-0)
Supplement: Supplementary file 1 — Supplementary Material 1. [file 12889_2025_24507_MOESM1_ESM.pdf]

Supplementary tables

Table 4: **Priors for Model Parameters by Country and Cluster.** The table summarizes prior distributions used for Bayesian inference. LN(μ, σ) denotes a LogNormal distribution and N(μ, σ) denotes a Normal distribution. All values are log-transformed for consistency.

Cluster	Country	γ	I_0	r	A	S_0	ξ
1	Cameroon	LN(1.32, 0.1)	LN(8, 0.1)	LN(4.33, 0.4)	LN(0.88, 0.08)	LN(5976.98, 0.1)	N(125.15, 5)
1	Comoros	LN(0.54, 0.3)	LN(8, 0.1), LN(10, 0.1)	LN(0.72, 0.2)	LN(0.60, 0.08)	LN(12220, 0.05)	N(10, 5)
1	Somalia	LN(0.76, 0.3)	LN(202, 0.1)	LN(0.20, 0.1)	LN(0.74, 0.08)	LN(29125, 0.1)	N(40, 5)
2	Malawi	LN(1.06, 0.5)	LN(560, 0.6)	LN(0.10, 0.1)	LN(0.82, 0.08)	LN(59874, 0.83)	N(1, 5)
2	Mozambique	LN(1.06, 0.5)	LN(560, 0.6)	LN(0.10, 0.1)	LN(0.82, 0.08)	LN(59874, 0.83)	N(1, 5)
3	Sudan	LN(0.90, 0.03)	LN(1, 0.1)	LN(0.40, 0.1)	LN(0.50, 0.07)	LN(14952, 0.08)	N(5, 3)
3	Zambia	LN(0.76, 0.3)	LN(98, 0.1)	LN(0.40, 0.1)	LN(0.82, 0.08)	LN(40199.00, 0.083)	N(50, 5)
3	Zimbabwe	LN(0.95, 0.5)	LN(256, 0.1)	LN(0.40, 0.1)	LN(0.82, 0.08)	LN(36470, 0.1)	N(78, 5)

Table 5: **Estimated parameter medians and 95% credible intervals for each country, grouped by cluster.** Parameters include: maximum infection rate (η), recovery rate (γ), bacteria intrinsic growth rate (r), and human bacteria shedding rate (ξ), all per week.

Cluster	Country	η	γ	r	ξ
1	Cameroon	0.93 (0.83, 1.00)	0.96 (0.88, 1.00)	0.92 (0.64, 1.24)	128.3 (119.3, 137.7)
1	Comoros	0.64 (0.55, 0.74)	0.60 (0.50, 0.83)	0.66 (0.46, 0.91)	22.56 (17.80, 27.97)
1	Somalia	0.65 (0.55, 0.76)	0.92 (0.74, 1.00)	0.24 (0.19, 0.29)	13.27 (9.89, 16.84)
2	Malawi	0.83 (0.71, 0.97)	0.43 (0.15, 0.87)	0.10 (0.08, 0.12)	1.75 (0.99, 2.98)
2	Mozambique	0.72 (0.63, 0.83)	0.71 (0.41, 0.98)	0.72 (0.58, 0.87)	32.03 (24.32, 40.68)
3	Sudan	0.93 (0.84, 0.99)	0.91 (0.90, 0.95)	0.29 (0.24, 0.34)	35.58 (31.91, 39.11)
3	Zambia	0.71 (0.62, 0.81)	0.89 (0.67, 1.00)	0.44 (0.36, 0.53)	35.77 (28.69, 43.39)
3	Zimbabwe	0.59 (0.49, 0.70)	0.97 (0.88, 1.00)	0.47 (0.39, 0.57)	19.36 (14.91, 24.93)

Table 6: **Estimated initial infected (I_0) and initial susceptible (S_0) individuals with 95% credible intervals, grouped by cluster.**

Cluster	Country	I_0	S_0
1	Cameroon	7.98 (6.95, 9.15)	6438.53 (5507.58, 7519.03)
1	Comoros	9.46 (8.20, 10.83)	12564.48 (11429.30, 13745.92)
1	Somalia	230.00 (186.00, 281.00)	17963.00 (15644.00, 21350.00)
2	Malawi	1054.50 (521.96, 1886.69)	56665.02 (43429.26, 74960.48)
2	Mozambique	8.81 (7.22, 10.67)	55546.95 (37122.22, 76132.92)
3	Sudan	1.00 (1.00, 1.00)	27704.00 (24572.00, 31247.00)
3	Zambia	90.00 (74.00, 109.00)	31178.00 (27080.00, 36200.00)
3	Zimbabwe	281.00 (229.00, 338.00)	39425.00 (34497.00, 44242.00)

Table 7: **Countries parameter median** The median is calculated with the median value of the parameters for all the countries

Country	Global median	Global SD	Global 2.5%	Global 95%
S	31012	18165	5997	68484
I	210	357	1	1311
r	0.48	0.27	0.09	1.05
γ	0.7	0.3	0.12	1
η	0.76	0.14	0.53	1
ξ	33.49	37.34	1.32	132.29

Table 8: **Socioeconomic, Environmental Indicators and Disaster Frequencies by Cluster.** GINI-Index [2] measures income inequality (0=perfect equality, 1=perfect inequality); HDI is the Human Development Index; GNI is Gross National Income in USD; EPI-DWS (%) represents access to safe drinking water from the Environmental Performance Index [47]; and Open Defecation (%) is the percentage of the population practicing open defecation [56]. Disaster frequency is the count of reported EM-DAT events (accessed via public.emdat.be/data [7]) per country that are associated with cholera transmission risk. This includes variants of water-related disasters—**Water**, **Flood (General)**, **Flash flood**, **Tropical cyclone**, **Bacterial disease (Cholera)**, **Landslide (wet)**, **Storm surge**, and **Severe weather**—as well as any events explicitly labeled "Cholera" in the dataset.

Cluster	Country	GINI-Index	HDI	GNI (USD)	EPI DWS (%)	Open Defecation (%)	Disaster Frequency
1	Cameroon	0.466	0.587	3681.47	23.8	4.3	8
1	Comoros	0.453	0.586	3260.56	26.9	0.6	2
1	Somalia	0.368	0.380	1072.20	25.6	21.3	8
2	Malawi	0.516	0.508	1432.47	17.8	2.6	8
2	Mozambique	0.540	0.461	1219.24	21.3	19.6	11
3	Sudan	0.342	0.516	3514.77	41.0	20.0	3
3	Zambia	0.570	0.569	3157.36	22.3	6.4	5
3	Zimbabwe	0.503	0.550	2078.92	19.7	17.3	5

Table 9: **Shapiro-Wilk Test Results for Parameters Across Clusters.** The Shapiro-Wilk test assesses the normality of parameter distributions within each cluster. P-values below 0.05 indicate a deviation from normality (*Not Normal*), while p-values above 0.05 suggest the data is normally distributed (*Normal*). Cluster 2 is marked as *Skipped* due to insufficient sample size (fewer than 3 observations), which prevents the Shapiro-Wilk test from being performed.

Parameter	Cluster 1	Cluster 2	Cluster 3
η	0.046 (Not Normal)	(Skipped)	0.601 (Normal)
γ	0.206 (Normal)	(Skipped)	0.156 (Normal)
r	0.738 (Normal)	(Skipped)	0.263 (Normal)
ξ	0.139 (Normal)	(Skipped)	0.394 (Normal)
S	0.931 (Normal)	(Skipped)	0.479 (Normal)
I	0.011 (Not Normal)	(Skipped)	0.609 (Normal)
Gini index	0.234 (Normal)	(Skipped)	0.554 (Normal)
HDI	0.008 (Not Normal)	(Skipped)	0.691 (Normal)
GNI	0.288 (Normal)	(Skipped)	0.461 (Normal)
EPI drinking water score	0.823 (Normal)	(Skipped)	0.214 (Normal)
Open defecation percentage	0.322 (Normal)	(Skipped)	0.361 (Normal)

Table 10: **Levene's Test Results for Homogeneity of Variances Between Clusters 1 and 3.** The Levene's test assesses whether variances of a parameter are homogeneous across clusters. P-values above 0.05 suggest homogeneous variances (*Homogeneous*), while p-values below 0.05 indicate a lack of homogeneity (*Heterogeneous*).

Variable	Levene's Test Statistic	Levene's Test p-value
Open defecation percentage	0.15	0.718 (Homogeneous)
EPI drinking water score	1.067	0.36 (Homogeneous)
GNI	0.278	0.626 (Homogeneous)
HDI	0.55	0.499 (Homogeneous)
GINI index	0.652	0.465 (Homogeneous)
I	0.043	0.845 (Homogeneous)
S	0.002	0.967 (Homogeneous)
ξ	0.844	0.41 (Homogeneous)
r	1.543	0.282 (Homogeneous)
γ	0.361	0.58 (Homogeneous)
A	0.089	0.78 (Homogeneous)

Table 11: **ANOVA and Kruskal-Wallis Test Results for Parameters Between Clusters 1 and 3.** P-values below 0.05 indicate statistically significant differences (*Significant*), while p-values above 0.05 suggest no significant difference (*Not Significant*).

Parameter	Test	p-value	Interpretation
r	ANOVA	0.374	Not Significant
S	ANOVA	0.012	Significant
γ	ANOVA	0.6	Not Significant
ξ	ANOVA	0.449	Not Significant
GINI INDEX	ANOVA	0.597	Not Significant
GNI	ANOVA	0.802	Not Significant
EPI_Drinking Water Score	ANOVA	0.758	Not Significant
Open Defecation Percentage	ANOVA	0.486	Not Significant
A	Kruskal-Wallis	0.827	Not Significant
I	Kruskal-Wallis	0.827	Not Significant
HDI	Kruskal-Wallis	0.513	Not Significant

Table 12: **ANOVA Results for Parameters Across Groups.** P-values below 0.05 indicate significant differences (*Yes*), while p-values above 0.05 indicate no significant differences (*No*).

Parameter	F-statistic	p-value	Significant	Median: Group 1	Group 2	Group 3
A	0.032	0.969	No	0.738	0.775	0.763
EPI Drinking Water Score	0.720	0.531	No	25.433	19.550	27.667
GINI Index	0.881	0.470	No	0.429	0.528	0.472
GNI	1.641	0.283	No	2671.409	1325.857	2917.014
HDI	0.360	0.717	No	0.518	0.485	0.545
I	1.119	0.397	No	82.624	531.659	124.467
Open Defecation (%)	0.262	0.780	No	8.733	11.100	14.567
S	43.365	0.001	**Yes**	12322.278	56105.985	32972.966
γ	0.419	0.679	No	0.829	0.566	0.666
r	0.435	0.670	No	0.605	0.407	0.398
ξ	0.623	0.573	No	54.716	16.890	23.328
